# Optical-flow based non-invasive analysis of cardiomyocyte contractility

**DOI:** 10.1038/s41598-017-10094-7

**Published:** 2017-09-04

**Authors:** Andras Czirok, Dona Greta Isai, Edina Kosa, Sheeja Rajasingh, William Kinsey, Zoltan Neufeld, Johnson Rajasingh

**Affiliations:** 1 Department of Anatomy & Cell Biology, University of Kansas Medical Center, Kansas City, KS USA; 2 Department of Biological Physics, Eotvos University, Budapest, Hungary; 3 Department of Mathematics, University of Queensland, Brisbane, Australia; 4 Department of Internal Medicine, University of Kansas Medical Center, Kansas City, KS USA

## Abstract

Characterization of cardiomyocyte beat patterns is needed for quality control of cells intended for surgical injection as well as to establish phenotypes in disease modeling or toxicity studies. Optical-flow based analysis of videomicroscopic recordings offer a manipulation-free and efficient characterization of contractile cycles, an important characteristics of cardiomyocyte phenotype. We demonstrate that by appropriate computational analysis of optical flow data one can identify distinct contractile centers and distinguish active cell contractility from passive elastic tissue deformations. Our proposed convergence measure correlates with myosin IIa immuno-localization and is capable to resolve contractile waves and their synchronization within maturing, unlabeled induced pluripotent stem cell-derived cardiomyocyte cultures.

## Introduction

Heart diseases are the leading cause of death in the United States and around the world^1^. Hypertension, tobacco exposure, high cholesterol, obesity, diabetes, unhealthy diet, alcohol, and aging seem to have an additive effect for the causation of cardiac diseases. Unlike other organs, cardiac tissue does not regenerate after injury. Thus, when cardiac tissue is damaged by obstruction to the blood supply, it forms a scar. Scar tissue is not functional, and if it occupies large part of the myocardium, the heart is unable to supply the metabolic needs of the human body. Currently, 5.7 million Americans are living with heart failure, and about 10% have advanced heart failure^2^. Since donor hearts are in short supply, cardiac repair with cell therapy has emerged as a promising treatment alternative in this patient population. Successful generation of induced pluripotent stem cells (iPSCs) and their differentiation into cardiomyocytes have created exciting possibilities^3–5^, to repair damaged myocardium.

Medical-grade, induced pluripotent stem cell (iPSC)-derived cardiomyocytes are promising both as implants to improve cardiac function and also as tools to model cardiac diseases. Myocardial tissue repair using iPSC-derived cardiomyocytes, reprogrammed from a patient’s somatic cells by mechanisms analogous to those taking place during embryonic development, is a particularly promising avenue for future treatment of cardiac diseases. Recently, we have formulated a combinatorial, safe, animal-free and viral-free approach using DNA and RNA pluripotent factors that can reprogram a wide range of adult human cells into iPSCs–with subsequent differentiation into functional cardiomyocytes^6^.

Recent studies have shown that iPSC-derived cardiomyocytes yield an adult phenotype through a maturation process^7, 8^. While these studies were primarily focused on electrophysiological end-points, the most important characteristic of a cardiomyocyte, however, is its ability to contract. Thus, quantifying contractility is essential for measuring the functionality of cardiomyocytes. Unlike most current technologies, optical flow-based (particle image velocimetry, PIV) methods^6, 9–11^ are capable of monitoring cardiomyocyte contractile function without physical or biochemical manipulations, hence without compromising cell quality.

By considering the mechanics of an elastic plate with an embedded contractile center, we propose novel image-processing tools to monitor and evaluate the contractility of reprogrammed, iPSC-derived cardiomyocytes in high cell density culture conditions. With the help of these computational tools, we are able to discriminate actively contracting cell clusters from cells undergoing passive, elastic deformation. We demonstrate that our contractility measure identifies cardiomyocyte clusters with high myosin expression, and it can track culture maturation by determining the extent of spatial synchronization.

## Results

### Optical flow-based displacement analysis

**Figure 1:**
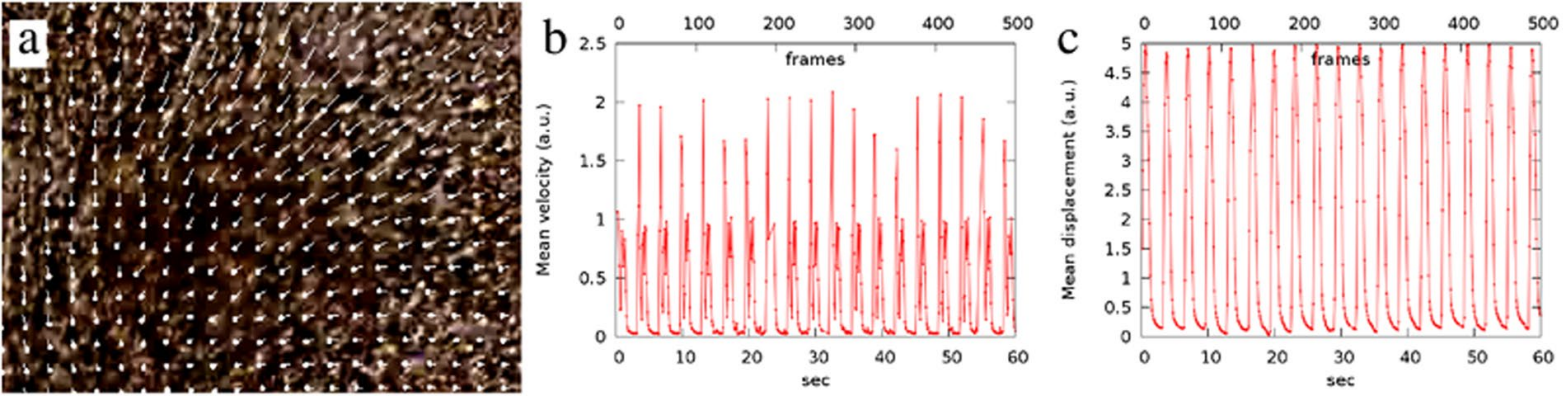
Beating cardiomyocytes, visualized by optical flow analysis of videomicroscopic images. (**a**) Optical flow-derived displacement vectors, superimposed on the corresponding bright field image of cardiomyocytes. (**b**) Velocity graph: the average magnitude of displacement vectors, calculated from consecutive image pairs. The sequence of double peaks corresponds to sudden contraction and slower, but more prolonged relaxation events. (**c**) The displacement graph (beat pattern) is calculated by comparing each image to an appropriate reference image.

To characterize cardiomyocyte function, we first apply our non-invasive, optical flow-based method to iPSC-derived cardiomyocyte cultures^6^ (Fig. 1a). Velocity graphs typically show a sequence of doublet peaks (Fig. 1b). Peaks indicating faster movement are associated with contraction and are followed by broader peaks corresponding to relaxation. Displacements, calculated relative to appropriate reference images, provide a signal that is simpler and less noisy than velocity graphs are. Such beat patterns thus represent myocardial contractile cycles with the rising and dropping impulse edges corresponding to contraction and relaxation, respectively. While contraction is usually swift, relaxation is slower and well approximated by an exponential function (Fig. 1c).

**Figure 2:**
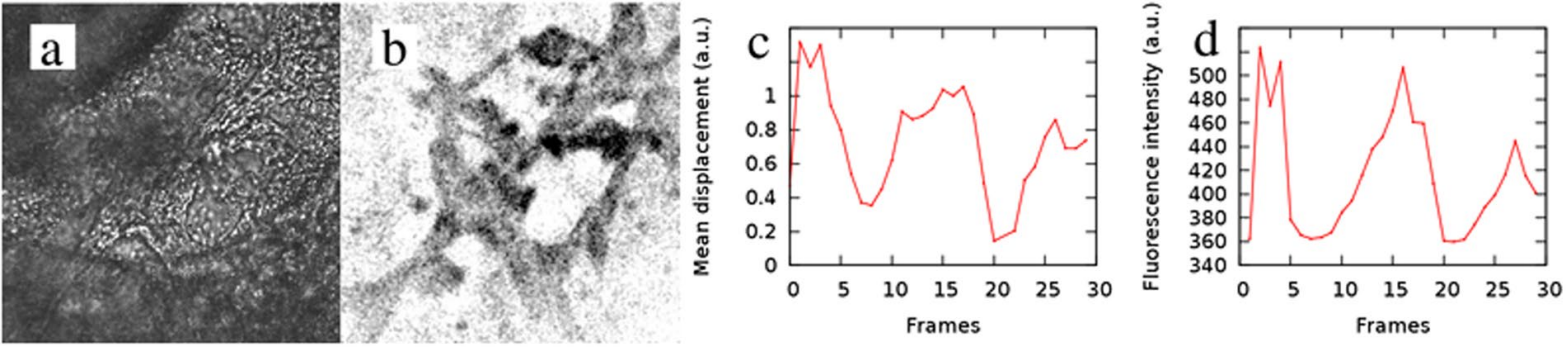
Comparison of an optical flow-derived beat pattern with Ca^++^ oscillations. Brightfield (**a**) and corresponding Ca^++^ (**b**) image of cardiomyocytes, recorded at a peak of the Ca^++^ signal. To improve contrast, Ca^++^ sensitive dye fluorescence image was inverted, thus darker areas indicate higher Ca^++^ concentration. Beat patterns (**c**) were obtained from optical flow analysis, and show a pattern similar to that of the Ca^++^ -dependent total fluorescence (**d**). Recording framerate: 1/s, field of view: 140 *μ*m.

We compared the optical flow-derived beat patterns with data obtained by a more conventional method, Ca^++^ imaging. Periodic Ca^++^ waves were visualized in iPSC-derived cardiomyocyte cultures by scanning confocal microscopy. The corresponding brightfield images, recorded by the auxiliary CCD camera of the confocal microscope, were analyzed by our optical flow software. The localization of Ca^++^ activity closely corresponds to the area where myocardial movements are detectable. Moreover, the temporal beat patterns obtained either from fluorescence intensity or from displacement magnitudes are highly similar (Fig. 2). Thus, our optical flow-based analysis is a reasonable non-invasive substitute for the more expensive and labor intensive traditional method to evaluate cardiomyocyte beat phenotypes.

### Mechanics of a contractile cell layer

While beat patterns are useful to represent the beating activity of a single contractile center within a microscopic field of view, they have limited utility to locate contractile centers or to characterize multiple contractile foci within the same microscopic field. High cell density cultures are a mechanical continuum, hence forces exerted at a certain location can deform and move distant cells. To deduct active cell contractility from the observed spatial distribution of displacement vectors, we consider the mechanics of a cardiomyocyte layer that undergoes elastic deformations driven by internal active cell contractility.

While cardiomyocytes are attached to a rigid culture substrate, the observed beat patterns indicate that the monolayer can deform sufficiently to accommodate even relatively large cell displacements. We thus envision that either (i) the cytosol of each cardiomyocyte can accommodate substantial shear between the basal cell cortex and the rest of the cell body, or (ii) cardiomyocytes participating in periodic contractile behavior lose their adhesion to the underlying rigid substrate. In our first, simplest approach, we treat the cardiomyocyte layer as an elastic plate, freely moving in two dimensions.

The force balance equation in the absence of external forces

1 $$\nabla \cdot ({\boldsymbol{\sigma }}+{{\boldsymbol{\sigma }}}^{\ast })=0$$

includes the elastic stress **σ** and the active contractile stress of the cells **σ** ^*^. Let us consider a single contractile center of radius *r*_0_ at the origin of a polar coordinate system (*r*, *ϕ*), thus **σ** ^*^ = 0 for *r* > *r*_0_. The elastic displacement **u** in polar coordinates is given by the radial and tangential components *u*_*r*_ and *u*_*ϕ*_, respectively. Since the system is rotation invariant, *u*_*r*_ = *u*(*r*) and *u*_*ϕ*_ = 0. For such a displacement field, the infinitesimal strain tensor

2 $${\boldsymbol{\varepsilon }}=\frac{1}{2}(\frac{\partial {\bf{u}}}{\partial {\bf{r}}}+{\frac{\partial {\bf{u}}}{\partial {\bf{r}}}}^{T}),$$

when expressed in polar coordinates, has the following components

3 $${\varepsilon }_{r,r}=\frac{du}{dr},\quad {\varepsilon }_{\varphi ,\varphi }=ur,\quad {\varepsilon }_{r,\varphi }=0.$$

While the passive elastic response of the myocardium is well described by an anisotropic and viscoelastic constitutive equation^12, 13^, for simplicity here we consider an isotropic linear elastic membrane. Then the elastic stress and the strain tensor is related through Hooke’s law as

4 $${\boldsymbol{\sigma }}={c}_{1}{\boldsymbol{\varepsilon }}+{c}_{2}{\bf{ITr}}({\boldsymbol{\varepsilon }}\mathrm{).}$$

where *c*_1_ and *c*_1_ characterize the elasticity of the cardiomyocyte sheet. Thus, for the components of the strain tensor **σ** we obtain

5 $${\sigma }_{r,r}={c}_{1}\frac{du}{dr}+{c}_{2}(\frac{du}{dr}+\frac{u}{r})$$

6 $${\sigma }_{\varphi ,\varphi }={c}_{1}\frac{u}{r}+{c}_{2}(\frac{du}{dr}+\frac{u}{r})$$

7 $${\sigma }_{r,\varphi }=0.$$

In the absence of active contractility (**σ** ^*^ = 0 for *r* > *r*_0_), according to Eq. (1) the condition for mechanical equilibrium is $\nabla \cdot {\boldsymbol{\sigma }}=0$. Expressed with polar coordinates,

8 $$\frac{d{\sigma }_{r,r}}{dr}+\frac{1}{r}({\sigma }_{r,r}-{\sigma }_{\varphi ,\varphi })=0.$$

Substituting expressions (5–7) into Eq. (8), for *u*(*r*) we obtain:

9 $$\frac{{d}^{2}u}{d{r}^{2}}+\frac{1}{r}\frac{du}{dr}-\frac{1}{{r}^{2}}u=0.$$

This is the Cauchy-Euler equidimensional equation. The solution of (9) satisfying the ${\mathrm{lim}}_{r\to \infty }u(r)=0$ boundary condition is *u*(*r*) = *a*/*r*, where *a* is a constant. Thus, using relations (5–7), the strain tensor in polar coordinates is given as

10 $${\boldsymbol{\sigma }}=(\begin{array}{cc}{\sigma }_{r,r} & {\sigma }_{r,\varphi }\\ {\sigma }_{r,\varphi } & {\sigma }_{\varphi ,\varphi }\end{array})=\frac{a{c}_{1}}{{r}^{2}}(\begin{array}{cc}-1 & 0\\ 0 & 1\end{array})\mathrm{.}$$

Calculating the trace of (4) yields

11 $${\bf{Tr}}{\boldsymbol{\sigma }}=({c}_{1}+2{c}_{2}){\bf{Tr}}({\boldsymbol{\varepsilon }}\mathrm{).}$$

According to Eqs (10) and (11) ${\bf{Tr}}{\boldsymbol{\sigma }}={\bf{Tr}}{\boldsymbol{\varepsilon }}=0$. As ${\bf{Tr}}{\boldsymbol{\varepsilon }}=\nabla \cdot {\bf{u}}$, for *r* > *r*_0_ we thus obtain

12 $$\nabla \cdot {\bf{u}}=0.$$

Conversely, near the contraction center, for *r* < *r*_0_ we assume a uniform active contraction of the cells:

13 $${{\boldsymbol{\sigma }}}^{\ast }={\sigma }_{\ast }{\bf{I}}\mathrm{.}$$

The condition for mechanical equilibrium (1) now also yields (8), with the boundary condition *u*(*r*) = 0. Substituting the corresponding linear solution *u*(*r*) = *br* with a parameter *b* into Hooke’s law (4) yields

14 $$\sigma =b({c}_{1}+2{c}_{2}){\bf{I}}\mathrm{.}$$

Substituting into (11) we obtain

15 $$\nabla \cdot {\bf{u}}={\bf{Tr}}{\boldsymbol{\varepsilon }}=2b=-\frac{2{\sigma }_{\ast }}{{c}_{1}+2{c}_{2}}\mathrm{.}$$

Since contractility implies negative divergence, we denote $C=-\nabla \cdot {\bf{u}}$ as *convergence*. By considering (12) and (15), we can see that for a freely floating cell sheet the convergence *C* of the displacement field **u** is thus a measure proportional to σ_*_, the active contractility of the cells.

A more realistic approach takes into account the elastic shear stress generated between the substrate-attached basal cytoskeletal layer and the rest of the cardiomyocyte cell body. Thus, the equation for mechanical equilibrium (1) is replaced by

16 $$\nabla \cdot ({\boldsymbol{\sigma }}+{{\boldsymbol{\sigma }}}^{\ast })-k{\bf{u}}=0$$

where *k* is the “spring constant” of the elastic shear force acting between the cell body and the substrate. For *r* > *r*_0_, the equation analogous to (9) becomes

17 $$({c}_{1}+{c}_{2})(\frac{{d}^{2}u}{d{r}^{2}}+\frac{1}{r}\frac{du}{dr}-\frac{1}{{r}^{2}}u)-ku=0.$$

Scaling the variable by $\lambda ={({c}_{1}+{c}_{2})}^{\mathrm{1/2}}{k}^{-\mathrm{1/2}}$ as *ρ* = *r*/*λ*, the drag coefficient *k* can be eliminated and (17) is transformed into the modified Bessel’s equation

18 $${\rho }^{2}\frac{{d}^{2}ud}{{\rho }^{2}}+\rho \frac{du}{d\rho }-({\rho }^{2}+\mathrm{1)}u=0.$$

**Figure 3:**
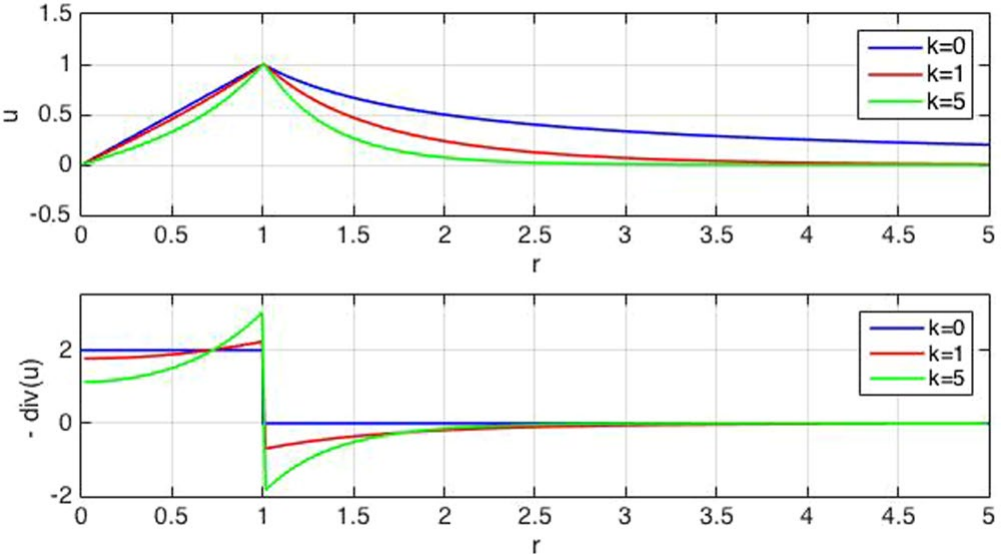
Deformations of an elastic sheet attached to a rigid substrate and actively contracted within a radius of *r*_0_ = 1. Radial displacement *u*(*r*) (top) and its convergence (negative divergence) $C=-\nabla \cdot {\bf{u}}$ (bottom) is shown for various substrate adhesion strengths *k* = 0, 1, 5.

Equation (18) has two linearly independent solutions. One of them grows, while the other decays exponentially as *r* → ∞. For *r* > *r*_0_ the suitable solution is the modified Bessel’s function of the second kind, *K*_1_(*ρ*), that decays at infinity. The equation for the deformation within the actively contracting core remains (18), but the condition *u*(0) = 0 is satisfied by the modified Bessel’s function of the first kind, *I*_1_(*ρ*). For small values of *ρ*, i.e. close to the center, *I*_1_(*ρ*) increases approximately proportionally with *ρ* (Fig. 3). The pre-factors of both *K*_1_ and *I*_1_ can be determined numerically by matching the displacement at the boundary of the two regions.

Figure 3 illustrates the effect of substrate adhesion on the displacement and convergence in case of a uniformly contracting core of size *r*_0_ = 1. Without adhesion, the deformation *u*(*r*) is proportional to *r* within the core, then decays as 1/r. The convergence of **u** is proportional to the active contractility, σ_*_, hence it drops to zero outside of the core region. With substrate adhesion the overall relationship between convergence and contractility remains similar, but corrections appear. The main correction is that immediately outside of the core region the convergence is negative and this divergent halo becomes more pronounced when the mechanical role of cell-substrate adhesion is stronger, i.e. for larger values of *k*. Displacements of the passive cells in the far field *r* ≫ *λ* can be calculated from the asymptotic approximation

19 $${K}_{1}(\rho ) \sim \sqrt{\frac{\pi }{2\rho }}{e}^{-\rho }\mathrm{.}$$

Thus, convergence decays exponentially outside of the contractile zone:

20 $$C=-\nabla \cdot {\bf{u}}=-\frac{1}{\rho }\frac{d\rho u(\rho )}{d\rho } \sim -\sqrt{\frac{\pi }{2\rho }}{e}^{-\rho }\mathrm{.}$$

Still, as Fig. 3 demonstrates, even for large *k* values localized contractility is identified by positive convergence.

**Figure 4:**
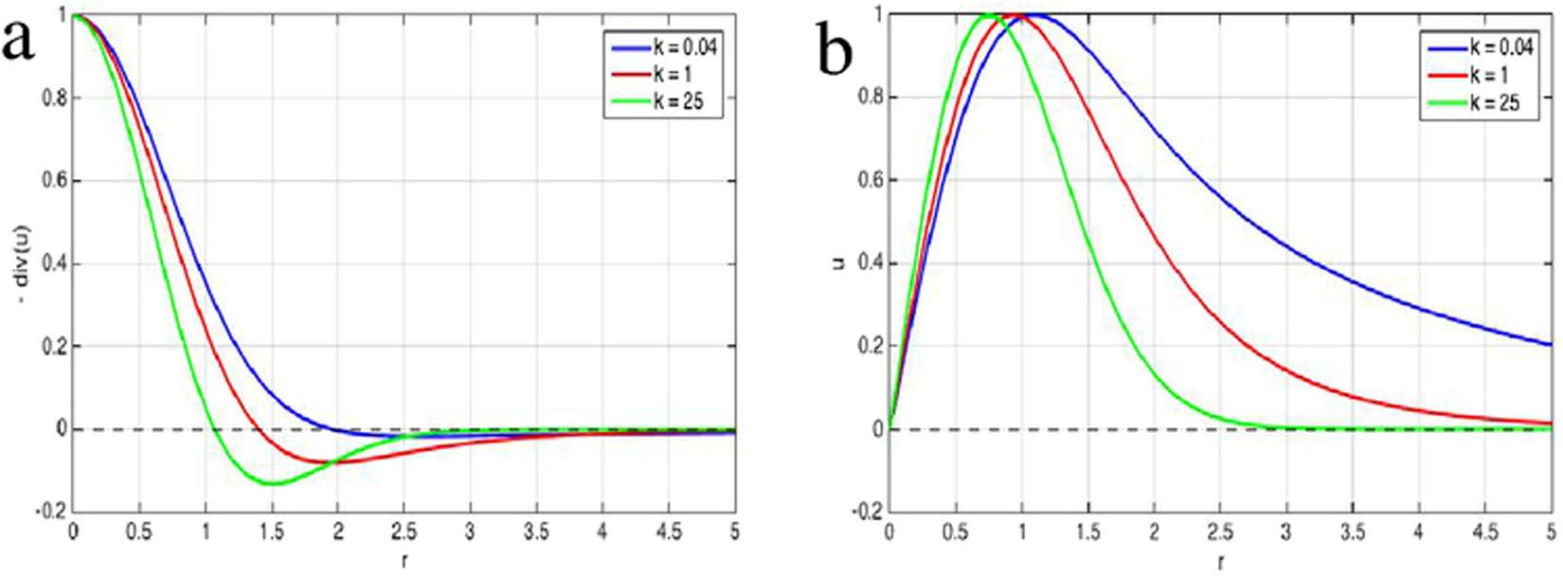
Radial profiles of convergence (**a**) and displacement (**b**) fields, when cell contractility changes in space gradually. Theoretical predictions were calculated by numerical integration of Eq. (21) assuming a Gaussian distribution of cell contractility centered at the origin. Convergence closely match local contractility for low values of *k* (blue). For increasing *k* an area with negative convergence appears at the boundary of the contractile region (red, green).

In the most general case of spatially non-uniform cell contractility (Fig. 4), we can write the displacement field as a convolution of contractility σ_*_ with the appropriate *K*_1_ solution of Eq. (18):

21 $${\bf{u}}({\bf{r}})=\int {\sigma }_{\ast }({\bf{r}}{\boldsymbol{^{\prime} }}){K}_{1}(\frac{|{\bf{r}}-{\bf{r}}{\boldsymbol{^{\prime} }}|}{\lambda })\frac{{\bf{r}}-{\bf{r}}{\boldsymbol{^{\prime} }}}{|{\bf{r}}-{\bf{r}}{\boldsymbol{^{\prime} }}|}d{\bf{r}}{\boldsymbol{^{\prime} }}\mathrm{.}$$

Hence, in general, the convergence is the convolution of the contractility and a response function *g*

22 $$C=-\nabla \cdot {\bf{u}}({\bf{r}})=\int {\sigma }_{\ast }({\bf{r}}{\boldsymbol{^{\prime} }})g(|{\bf{r}}-{\bf{r}}{\boldsymbol{^{\prime} }}|)d{\bf{r}}{\boldsymbol{^{\prime} }}$$

where

23 $$g(r)=\frac{1}{2\lambda }[{K}_{1}(\frac{r}{\lambda })-{K}_{2}(\frac{r}{\lambda })]\mathrm{.}$$

**Figure 5:**
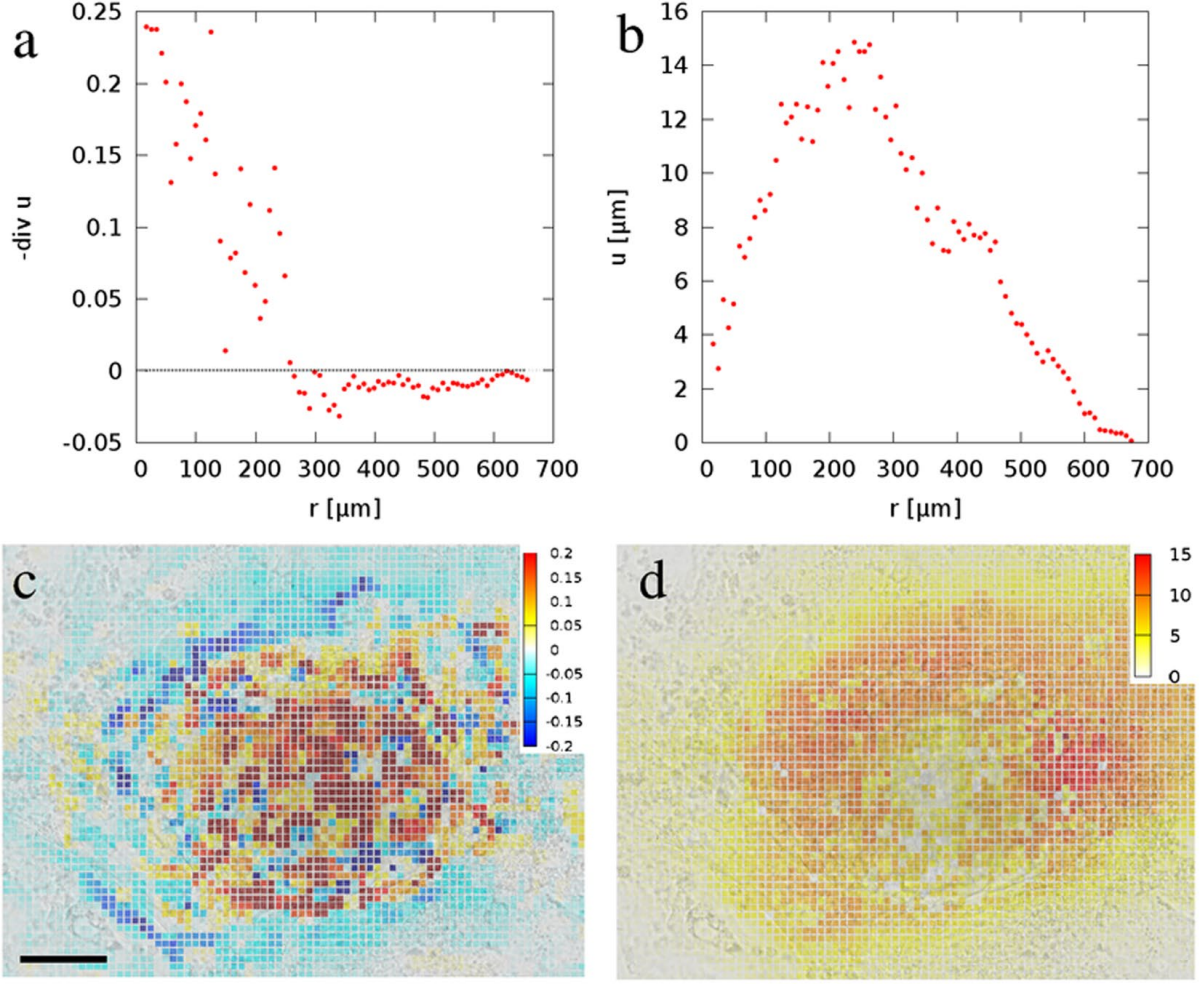
Experimental convergence (left) and displacement (right) data from iPSC-derived cardiomyocytes. The features predicted in Fig. 4 well characterize the radial convergence (**a**) and displacement (**b**) profiles, obtained by radial averaging of the corresponding convergence (**c**) and displacement (**d**) maps. Scale bar: 150 *μ*m.

In Fig. 5 we compare empirically obtained cardiomyocyte convergence and displacements fields with numerical predictions (Fig. 4) obtained using Eqs (21–23). We assumed that cell contractility is distributed according to a Gaussian function with radial symmetry around the origin, i.e., there is a gradual transition from actively contractile cells to passive cells. Note, that in this case the sharp discontinuity in the convergence seen in Fig. 3 is replaced by a smooth transition. Nevertheless, the positive convergence within the contractile region is still surrounded by a region of negative convergence as a result of the cell-substrate adhesion.

### Convergence analysis

**Figure 6:**
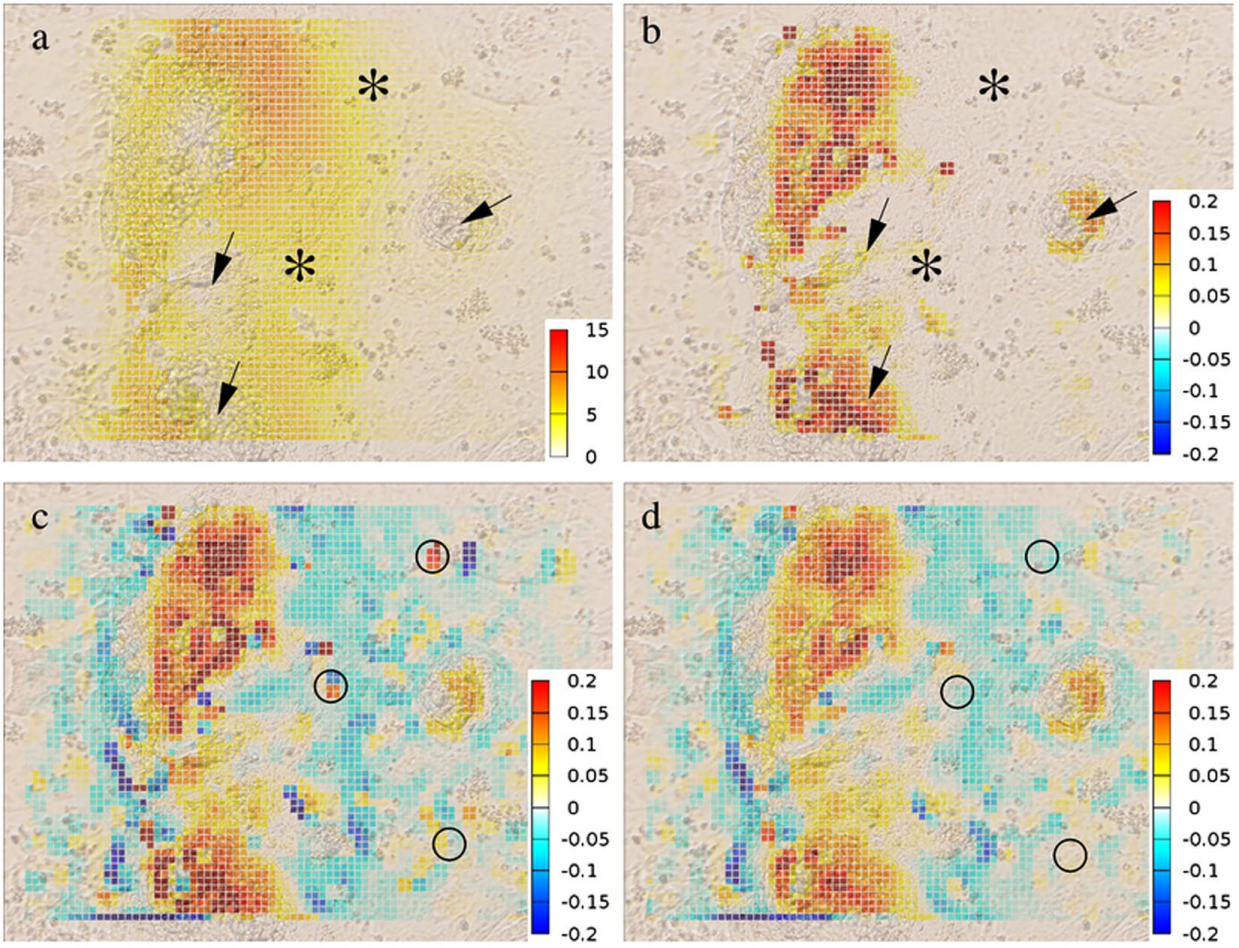
Contractility analysis using filtered convergence maps. An optical flow-derived displacement (**a**) and the corresponding contractility (**b**) field, calculated from frames of *in vitro* time-lapse imaging of cardiomyocytes. Warmer colors correspond to higher displacement or contractility magnitudes, respectively. The convergence field (**c**) is obtained from a time series by spatio-temporal filtering to remove various artifacts marked by circles (**d**). The procedure localizes contraction centers (red), which often do not correspond to areas with high displacement values (arrows). Conversely, cell layers often move passively without actively contracting (asterisks). Thus, convergence analysis provides information not readily available from velocity or displacement data.

Motivated by the above analysis, we calculated the convergence *C* from optical flow data to identify active contractile centers in iPSC-derived cardiomyocytes. As a numerical derivative, convergence is a noisy quantity. The strongest sources of noise are–when present–the cell-free areas within the analyzed microscopic field of view. In such areas the low contrast of the image cannot suppress non-biological movements, like debris moving with a convective flow of the medium. As such movements are rather quick, they yield a strong, rapidly changing signal in the optical flow analysis. A second source of noise is the slow accumulation of changes between the analyzed and the reference image frames. As cells continuously rearrange, relaxed states of the contractility cycles will not match precisely. This background noise increases with the time lag between the analyzed and reference frames. To suppress noise from both sources, we apply a (i) spatial median filter, a (ii) temporal high-pass filter and (iii) use contrast-weighted convergence values (Fig. 6). The combination of these approaches greatly reduces the computational noise and the noise-filtered convergence analysis efficiently identifies contraction centers. Finally, for simplicity our estimate of (relative) contractility *s* ^*^ is the thresholded convergence:

24 $${s}^{\ast }({\bf{r}})=\{\begin{array}{ll}C({\bf{r}})=-\nabla \cdot {\bf{u}}({\bf{r}}) & {\rm{for}}\,C({\bf{r}}) > 0\\ 0 & {\rm{otherwise}}{\rm{.}}\end{array}$$

**Figure 7:**
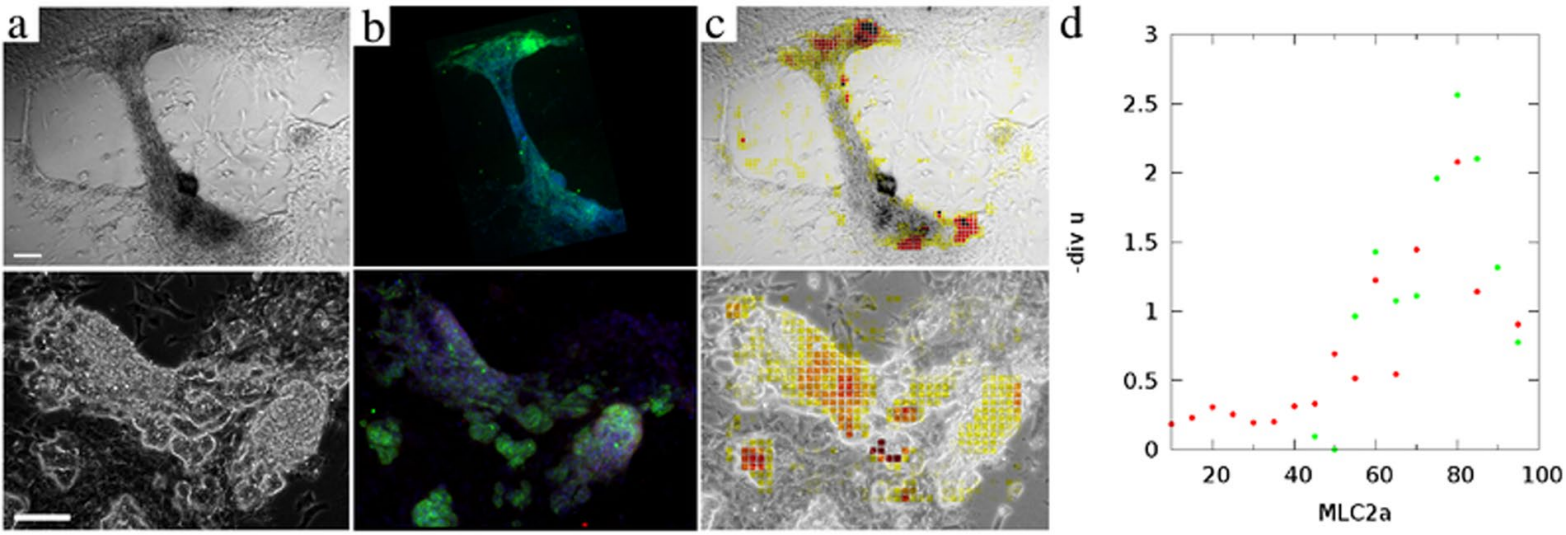
Contractile centers, identified by convergence analysis, co-localize with areas where myosin II expression is high. (**a**) Representative frames from brightfield videomicroscopy recordings, obtained from distinct cardiomyocyte cultures. Scale bars: 150 *μ*m. (**b**) Immunofluorescence localization of atrial Myosin IIa (green). (**c**) Convergence-estimated contractility *s* ^*^ (yellow to red colors), superimposed on brightfield micrographs. (**d**) Scatter plot of myosin IIa immunofluorescence intensity vs convergence-derived contractility for non-overlapping image tiles of the two independent samples.

To test the biological relevance of our analysis, we compared the results of convergence analysis with the distribution of myosin IIa within the same sample. Videomicroscopy recordings of cardiomyocyte cultures were followed by fixing the samples and immunostaining them against the myosin II variant found in atrial cells, the expected main source of contractile activity in our iPSC-derived cardiomyocyte cultures (Fig. 7). As the fixed and live specimen do not overlap precisely, to quantitate the degree of covariance we distributed both the convergence maps and the immunofluorescence images into tiles, and measured the total convergence and the total myosin IIa immunofluorescence intensity within each tile. The scatter plot of the corresponding convergence and myosin IIa values indicate a strong correlation between the two quantities, hence supporting our analysis. (Fig. 7d).

### Dynamics of contractile centers

**Figure 8:**
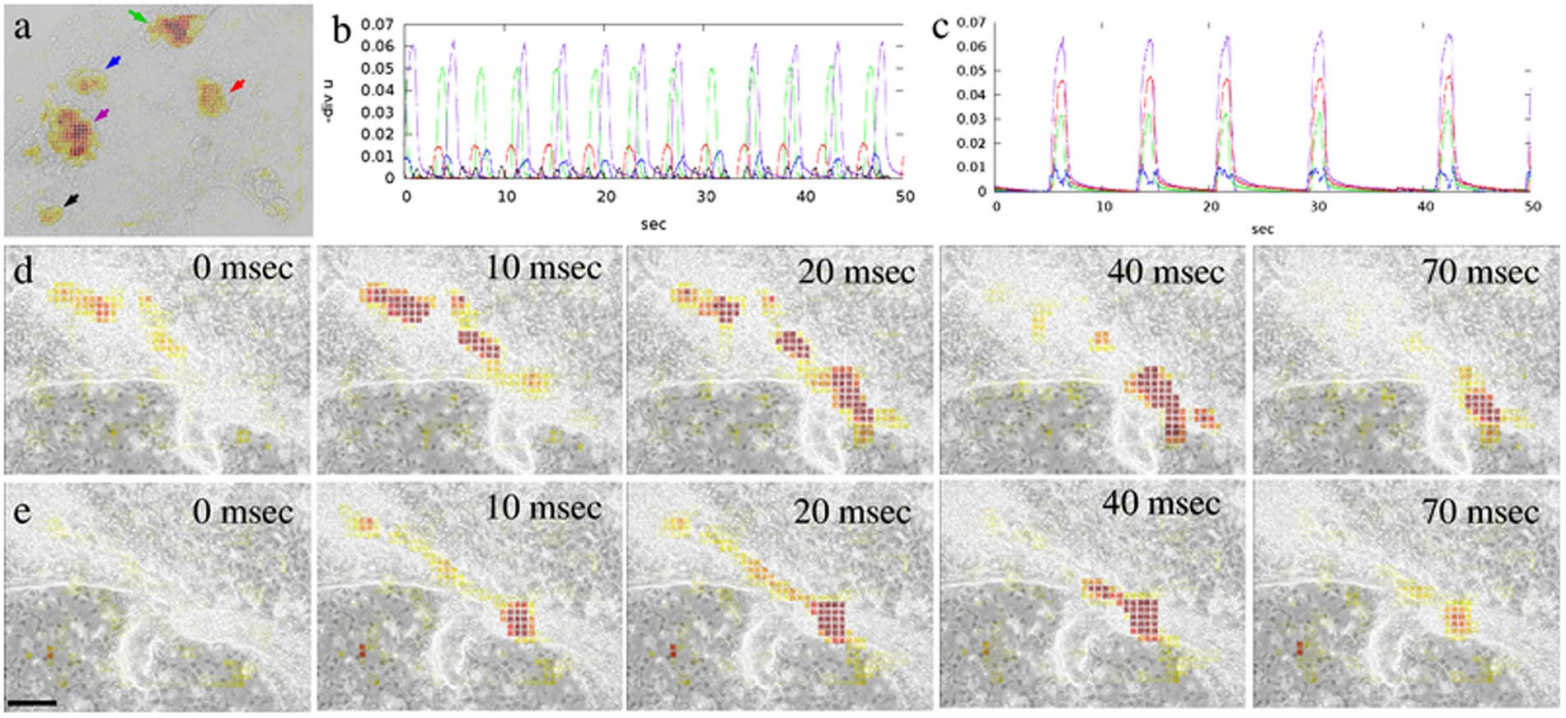
Synchronization of contractile centers. (**a**) Convergence analysis identifies distinct contractile centers (arrows) within a microscopic field recorded at day *in vitro* (DIV) 7. (**b**,**c**) The temporal activity of each contractile center is visualized by beat patterns obtained as the local integral of the convergence field. The color of the beat pattern graphs in panels b,c matches the color of the corresponding marker in panel a. At DIV 7 (panel b), centers marked with the red, green, blue and violet colors beat with the same frequency, but exhibit progressively delayed phases. The area identified by black color beats with a higher frequency. Unsynchronized contractile centers at DIV 7 (**b**) become fully synchronous 4 days later (**c**). (**d**) Contractile wave propagation is resolved at a 10/s frame rate in a DIV 10 cardiomyocyte culture. (**e**) One day later, the same area exhibits synchronous contractility. Scale bar: 150 *μ*m.

The proposed computational method allows us to study the formation and long-term behavior of beating cardiomyocyte nodes. Typically, early beating centers are not synchronized within a single microscopic field of view and beat at distinct frequencies which also frequently change in time (Fig. 8a,b). A sequence of convergence maps, calculated from high framerate recordings, can also reveal contractile waves passing through adjacent contractile centers (Fig. 8c). Within a day, however, cardiomyocyte contractility becomes synchronized in the entire field of view (Fig. 8c,e), even if contractile centers are separated by a barrier of non-beating cells.

## Discussion

Induced pluripotent stem cell (iPSC)-derived cardiomyocytes are expected to be useful in direct medical applications like restoring cardiac function in myocardial infarct patients^3–5^ or in fatal heart diseases^14, 15^. iPSC-derived cardiomyocytes are also likely to play a role in disease modeling. For most common diseases there are hundreds of genetic risk variants–each variant yielding a small change in phenotype. The variability of genotypes in such multi-factor diseases currently precludes proper risk evaluation as well as treatments optimized for the individual patients^16^. Phenotypes of iPSC-derived cardiac cells with various genetic backgrounds can be evaluated within an artificial tissue environment of varying complexity^17, 18^. Such experiments are expected to provide molecular disease mechanisms^19, 20^ and specific pharmacological intervention targets^21–23^. An optimal drug treatment regimen can be established for a certain individual patient or to treat rare diseases. Finally, iPSC-derived cardiomyocytes are also promising candidates for high throughput cardiotoxicity screens^24^. All these efforts require sensitive methods to evaluate cardiomyocyte function–both for quality control, and to establish functional aspects of cardiomyocyte phenotype.

Accordingly, several techniques are being used to characterize the most important cardiomyocyte function: contractility. Voltage and Ca^++^ sensitive dyes can reveal the electric and ionic waveforms that drive contractile cycles in a large number of cells^25, 26^. Patch clamping techniques allows detailed investigations, on the individual ion channel level, within a small cell population^27, 28^. Traction force microscopy can probe the forces cardiomyocytes transmit to an elastic substrate^29^. Cell shape changes associated with contractility can also be detected using electrical resistance measurements^30^. Videomicroscopy techniques are especially promising for high throughput contractility screens as they are simple, cheap and do not require manipulation of the cells. As the pioneering study of Hayakawa *et al*.^31^ established, optical-flow based techniques offer a useful estimate for the temporal dynamics of cardiomyocyte contractility *in vitro*. Accordingly, optical-flow has been used in several recent studies to characterize contractile cycles^6, 9–11, 32^. Here we demonstrate that by additional image processing steps, from the optical flow data one can obtain a both biologically and mechanically relevant *spatial map* of cell contractility.

The divergence of a vector field in two dimensions gives the net flux passing through the perimeter of a small area. A positive or negative divergence value thus indicates the presence of a source or sink at that position, respectively. Thus, contracting tissues display negative divergence, which we termed *convergence*. Our mechanical analysis gives more rigorous support to this intuitive argument, and identifies important corrections to this measure. We explored the feasibility to identify contractile cardiomyocytes as areas with positive convergence. In the future, more sophisticated methods may improve the sensitivity of the technique by solving the inverse convolution problem of Eqs (22 and 23). Thus, one can determine what is the most likely value of parameter *λ* and the most likely spatial map of cell contractility σ^*^ that is compatible with the experimentally observed convergence pattern *C*. A similar problem arises in traction force microscopy, where various deconvolution techniques have been utilized^33–35^. We expect the same techniques, like blind deconvolution or Wiener filtering, can also be helpful to achive higher resolution contractility maps. Better optical resolution would allow the 3D reconstruction of both the structure (height variation) of the cell culture and the cytoskeletal movements. Such a high resolution optical flow data, with corresponding finite element modelling^36^ would yield further insights into the mechanics of cardiomyocyte maturation.

The imaging technology we use to assay cardiomyocyte differentiation is a major departure from the traditional readouts which involve sophisticated instruments, and require either electrode clamping, using Ca^++^ -sensitive intracellular dyes or mechanically poking the cells. Each of these techniques is labor-intensive, invasive and thus can affect the vitality of the cells and their ability to produce contractile forces. Yet, the most important functional property of cardiomyocytes is the ability to produce contractile forces. Therefore, our ability to analyze and quantify cardiomyocyte contraction from microscopic images–without compromising cell quality–is a powerful assessment tool to monitor cardiomyocyte maturation. In the future, the behavior of disease-specific cardiomyocytes evaluated with this safe, manipulation-free optical method will also enable high-throughput drug screening and disease-specific drug discovery.

## Methods

### Data availability

The computational codes used in this paper are available at https://github.com/aczirok/piv-div, together with the appropriate image sequences at http://osf.io/2w63u.

### Cardiomyocyte culture

In a recent report^6^ we described an efficient method to generate human cardiomyocyte progenitor cells by reprogramming adult somatic cells such as skin fibroblasts. An induced pluripotent cell (iPSC) state is achieved by a combined treatment of pluripotent gene (Oct4, Nanog, Sox2 and Lin28) DNA and mRNA. The treatment regimen transformed 4fibroblasts into iPSC colonies, a significantly improved efficiency compared with use of either DNA or mRNA alone. These iPSC colonies were characterized by their mRNA and protein expressions. Human iPSCs were further differentiated into cardiomyocyte lineage in Matrigel coated culture dishes in the presence of a GSK inhibitor. Basic FGF was present initially for two days, but was replaced by a Wnt inhibitor for the subsequent five days. We observed the beating of cardiomyocytes in culture from day six onwards, and more than 85% of cells were positive for cardiotroponin T and Nkx2.5–measured by flow cytometry and immuno-staining. For the studies we used iPSC derived cardiomyocytes, after 15–20 days of maturation.

### Imaging

High framerate (10 frames/sec) movies were recorded using a cooled digital CCD camera (QImaging Retiga-SRV) mounted on a computer-controlled inverted microscope (Leica DMIRE2), equipped with a motorized stage. We used 5x and 10x objectives, both in phase contrast and brightfield modes.

Intracellular free Ca^++^ was measured in cells plated in dishes with glass-coverslip bottoms (TPG dish, ThermoFisher, Waltham, PA) and pre-incubated in medium containing a mixture of 10 *μ*M calcium green-AM in 0.4% pluronic F127 (ThermoFisher) for 30 min. Cells were then washed to remove unincorporated calcium green, and imaged with a 40X objective of a Nikon TE2000U confocal microscope, fitted with a temperature controlled enclosure maintained at 37 °C. Images were obtained at 1 second intervals with a 488 nm Spectra Physics (Mountain View, CA) laser. Emitted fluorescence was recorded with a 515/30 nm band pass filter and transmitted light was collected separately to obtain a bright field image.

### Optical flow analysis of image pairs

A motion pattern (velocity field) captured on a pair of images was calculated using the method described in refs 37, 38. Briefly, the first image was divided into overlapping square tiles, each 64 pixels wide. The displacement of each tile was determined by cross-correlation analysis: the second image was scanned pixel-by-pixel, by shifting an equally sized (64 pixels wide) square window for a location which exhibits a pattern most similar to the tile within the first image. The scanned area was centered at the position of the tile and allowed for 32 pixel displacements in each directions.

The similarity of two image tiles was quantified by the value of their cross correlation: the pixel-by-pixel sum of the product *h*_1_(*x*)*h*_2_(*x*) where *h*_1_(*x*) and *h*_2_(*x*) denote the brightness of corresponding pixel *x* within the original tile of the first image and within the window positioned on the second image, respectively. The most similar tile on the second image was then assumed to be the location to where the pattern in the first image moved to.

The displacement vectors characterizing each image tile were then interpolated and de-noised by a thin-plate spline fit, yielding our coarse displacement field. The coarse estimate was used to construct a second, higher resolution displacement field. In this second step, the cross-correlation search for pattern similarity was repeated with tiles that were only 32 pixels wide but in a much smaller search area allowing only for 4 pixel displacements around the location predicted by the coarse displacement field.

### Velocity field

To characterize the beating activity of cardiomyocytes, we analyzed 30 or 60 sec long video recordings. Each consecutive frame pair was subjected to optical flow analysis. The resulting vector field *v*(*t*, *x*) characterizes the average cell movement (speed and directionality) near pixel *x* at time *t*. For each time point *t*, the average motility *V*(*t*) was calculated as the spatial average of the speed magnitudes |*v*(*t*, *x*)| as

25 $$V(t)={\langle |v(t,x)|\rangle }_{x}\mathrm{.}$$

### Finding consecutive minima and maxima of a noisy signal

We assign an alternating sequence of minima ${t}_{k}^{min}$ and maxima ${t}_{k}^{max}$ to a signal *a*(*t*), such that for each *k*

26 $${t}_{k}^{min} < {t}_{k}^{max} < {t}_{k+1}^{min}$$

holds. The procedure filters out noisy extrema by requiring that $a({t}_{k}^{max})$ is the absolute maximum between ${t}_{k}^{min}$ and ${t}_{k+1}^{min}$, and $a({t}_{k+1}^{min}) < a({t}_{k}^{max})-{\rm{\Delta }}$, where Δ is a suitably chosen threshold. Similarly, we require that $a({t}_{k}^{min})$ is the absolute minimum in the interval $({t}_{k-1}^{max},{t}_{k}^{max})$, and $a({t}_{k}^{max}) > a({t}_{k-1}^{min})+{\rm{\Delta }}$.

### Beat patterns

Based on the average motility data, *V*(*t*), we determine a reference frame, which is a frame between two contraction cycles, where movement is minimal. Thus, the reference frame *t* = *t* ^*^ is a minimum of *V*(*t*).

Next, we perform optical flow analysis between each frame *t* and the reference frame *t* ^*^ yielding displacement vectors ${u}_{t\ast }(x,t)$. These displacement vectors estimate for each image frame *t* and location *x* the total movement (magnitude and directionality) relative to a resting (contraction-free) state. Similar to the average motility *V*(*t*), for each time point *t* the average displacement ${U}_{t\ast }(t)$ was calculated as the spatial average of the displacement magnitudes as

27 $${U}_{t\ast }(t)={\langle |{u}_{t\ast }(t,x)|\rangle }_{x}\mathrm{.}$$

We define the beat pattern *U*(*t*) as a signal synthesized using a sequence of optimal frames as references as

28 $$U(t)=\mathop{{\rm{\min }}}\limits_{k}{U}_{{t}_{k}^{min}}(t),$$

where the sequence ${t}_{k}^{min}$ were obtained as the minima of the ${U}_{t\ast }(t)$ signal. To reduce the computational load, *u*_*T*_(*t*, *x*) is often approximated as

29 $${u}_{T}(t,x)\approx {u}_{t\ast }(t,x)-{u}_{t\ast }(T,x\mathrm{).}$$

Our ability to resolve beat patterns is limited by the imaging frame rate. For frequencies higher than 120 bpm the 10 frame/sec image capture rate becomes inadequate. Beating frequency is established by assigning minima and maxima to the *U*(*t*) beat pattern, and calculating the mean period length between consecutive maxima.

### Convergence analysis

The optical flow-based method does not distinguish between active contractility and the passive, elastic response of the adjacent cell layer. To identify contractile centers, we estimated the convergence of the displacement field as its negative divergence:

30 $$\begin{array}{rcl}C({\bf{r}},t) & = & -\nabla \cdot {\bf{u}}({\bf{r}},t)=-(\frac{\partial {u}_{x}}{\partial x}+\frac{\partial {u}_{y}}{\partial y})\\  & \approx  & \frac{{u}_{x}(x-d,y,t)-{u}_{x}(x+d,y,t)}{2d}+\frac{{u}_{y}(x,y-d,t)-{u}_{y}(x,y+d,t)}{2d},\end{array}$$

where *d* is the resolution of the optical flow-derived grid.

### Noise reduction

#### Median filter

Local convergence data at each grid point was replaced by the median of the values taken from adjacent grid points. This nonlinear operation reduces noise while preserves spatial gradients^39^.

#### High-pass filter

A high pass filter, which attenuates low-frequency signal components, is ideal to remove the effects of the slow, cumulative changes in the non-contractile configuration while preserving the details of the much faster contraction-relaxation cycles. At each grid point **r** we thus replace the convergence *C*(**r**, *t*) with the filtered values *C′*(**r**, *t*) as

31 $$C^{\prime} ({\bf{r}},t)=q[C^{\prime} ({\bf{r}},t-\mathrm{1)}+C({\bf{r}},t)-C({\bf{r}},t-\mathrm{1)}]+\mathrm{(1}-q)M({\bf{r}}),$$

where 0 < *q* < 1 is a parameter controlling the cut-off frequency of the filter, and *M*(**r**) is the average of the unfiltered sequence *C*(**r**, *t*). The filter (31) thus maintains the average of a stationary sequence *C*(**r**, *t*).

#### Local contrast weighting

Areas devoid of cells can contribute noise to the optical flow analysis. As their image contrast is low, the optical flow algorithm easily picks up spurious correlations, or tracks debris floating with the medium. These effects can be suppressed by weighting the convergence data *C*(**r**, *t*) with the local image contrast *w*(*r*, *t*) as

32 $$C^{\prime} ({\bf{r}},t)=C({\bf{r}},t)w({\bf{r}},t)$$

where 0 ≥ *w* ≥ 1 is the local standard deviation of image brightness, calculated within a 16 × 16 pixel area, as described in ref. 40.
